# Horse ooplasm supports *in vitro* preimplantation development of zebra ICSI and SCNT embryos without compromising YAP1 and SOX2 expression pattern

**DOI:** 10.1371/journal.pone.0238948

**Published:** 2020-09-11

**Authors:** Andrés Gambini, Matteo Duque Rodríguez, María Belén Rodríguez, Olinda Briski, Ana P. Flores Bragulat, Natalia Demergassi, Luis Losinno, Daniel F. Salamone

**Affiliations:** 1 Facultad de Agronomía, Cátedra de Producción Equina, Universidad de Buenos Aires, Buenos Aires, Argentina; 2 Consejo Nacional de Investigaciones Científicas y Técnicas, Buenos Aires, Argentina; 3 Facultad de Agronomía, Cátedra de Fisiología Animal, Universidad de Buenos Aires, Buenos Aires, Argentina; 4 Facultad de Agronomía y Veterinaria, Cátedra de Producción Equina, Universidad Nacional de Río Cuarto, Río IV, Córdoba, Argentina; 5 Fundación Temaikèn, Belén de Escobar, Buenos Aires, Argentina; Faculty of Animal Sciences and Food Engineering, University of São Paulo, BRAZIL

## Abstract

Several equids have gone extinct and many extant equids are currently considered vulnerable to critically endangered. This work aimed to evaluate whether domestic horse oocytes support preimplantation development of zebra embryos obtained by intracytoplasmic sperm injection (ICSI, zebroid) and cloning, and to study the Hippo signaling pathway during the lineage specification of trophectoderm cells and inner cell mass cells. We first showed that zebra and horse sperm cells induce porcine oocyte activation and recruit maternal SMARCA4 during pronuclear formation. SMARCA4 recruitment showed to be independent of the genetic background of the injected sperm. No differences were found in blastocyst rate of ICSI hybrid (zebra spermatozoon into horse egg) embryos relative to the homospecific horse control group. Interestingly, zebra cloned blastocyst rate was significantly higher at day 8. Moreover, most ICSI and cloned horse and zebra blastocysts showed a similar expression pattern of SOX2 and nuclear YAP1 with the majority of the nuclei positive for YAP1, and most SOX2+ nuclei negative for YAP1. Here we demonstrated that horse oocytes support zebra preimplantation development of both, ICSI and cloned embryos, without compromising development to blastocyst, blastocyst cell number neither the expression of SOX2 and YAP1. Our results support the use of domestic horse oocytes as a model to study *in vitro* zebra embryos on behalf of preservation of valuable genetic.

## Introduction

Domestic horses and donkeys, wild asses, zebras and Przewalski’s horses share the only extant genus of the family Equidae: the genus *Equus*. Interestingly, individuals from the genus *Equus* have a remarkable genetic plasticity evidenced by the production of viable hybrids and the ability of mares, jennies and mules to carry on interspecific gestations [1–5]. Although mules (female horses x male donkeys) and hinnies (female donkey x male horse) are the best known equid hybrids, zebras (2n = 32 to 44) and Przewalski’s horses (2n = 66) are able to produce live offspring by intercross with domestic horses (2n = 64) and donkey (2n = 62) [6].

Over the last few centuries, several equine subspecies have gone extinct and many extant equids are considered vulnerable to critically endangered (IUCN 2020, http://www.iucnredlist.org/). Efforts for the preservation of wild equids are needed for the maintenance of animal population and genetic variability among individuals. To accomplish these goals, in addition to natural breeding, conservation programs can be enhanced by using assisted reproductive techniques (ART) to achieve optimal genetic management of endangered species and overcome infertility issues [7, 8]. Among ART, cryopreservation of gametes combined with *in vitro* embryo production are powerful tools for rescuing endangered animals or to preserve the genetics of critically endangered species. As conventional *in vitro* fertilization is not yet robust in horses, intracytoplasmic sperm injection (ICSI) and somatic cell nuclear transfer (SCNT) are the main techniques to produce *in vitro* embryos in horses that can be successfully cryopreserved for genetic banking and later embryo transfer [9]. ICSI in horses is on the front line relative to other domestic species, and it has become a widespread procedure for clinical uses [10] whereas developmental rates after ICSI in other domestic species are still low [11]. ICSI horse embryos have been obtained using different sources of semen, such as fresh, refrigerated, frozen, re-frozen, sex-sorted and lyophilized ejaculate [12–15] and also using epididymal frozen semen [16]. Although hybridization within equids is well known *in vivo*, to the best of our knowledge, there are no reports on *in vitro* production of hybrid equid embryos through ICSI. This could be a powerful tool to generate knowledge about the fertilization, genetics and early embryo development processes in these species. In parallel, the number of cloned horses produced by somatic cell nuclear transfer (SCNT) has also significantly increased over the last few years since the first cloned horse [17]; however, cloning efficiency remains poor [18].

The process of cell allocation during preimplantation embryo development has not been deeply investigated in equids. During the cell-fate allocation, SOX2 [SRY (Sex Determining Region Y) -box 2], one of the earliest known unique markers of inner cell mass (ICM) progenitors, is regulated by members of the HIPPO signaling pathway, including YAP1 (Yes Associated Protein 1) [19]. Moreover, early cell differentiation pathways during preimplantation embryo development, such as the HIPPO signaling pathway, could be compromised if chromatin-remodeling processes do not occur properly. During the maternal-to-zygotic transition, chromatin remodeling plays an essential role [20, 21]. The ATP-dependent chromatin remodeler SMARCA4 (Brahma-related gene 1, BRG1) translocates to the pronuclei soon after fertilization [22], and its mislocalization or reduction alters the regulation of transcription, RNA processing, and the cell cycle, leading to poor embryo development in mice [23–25].

Understanding the reproductive biology and generating comparative knowledge across species is essential to design and execute species-specific ART for animal conservation programs [7]. Although vast progress has been made in ART for the domestic horse and a select group of wild equids (Persian onager and Przewalski’s horse), there is no information on preimplantation embryo development in wild equids. Therefore, the aims of this study were: 1) to first analyze the ability of zebra (*Equus quagga burchelli*) sperm to trigger egg activation and SMARCA4 recruitment during pronuclear formation after ICSI, 2) to assess preimplantation development as well as SOX2 and YAP1 expression in hybrid (zebroid) ICSI embryos produced with domestic horse (*Equus ferus caballus*) oocytes as recipient for zebra sperm and, 3) to evaluate preimplantation development and the expression of SOX2 and YAP1 of interspecies SCNT zebra embryos produced with domestic horse oocytes as recipient for zebra somatic cells.

## Materials and methods

Unless otherwise stated, all chemicals were obtained from Sigma, St. Louis, MO, USA.

### Experimental design

#### Experiment 1

Due to the very limited access to horse oocytes, a porcine model was used for this experiment as a method to estimate the ability of zebra sperm to induce activation as it was reported previously for horses [26]. We compared the ability of zebra, horse and porcine sperm to activate and induce pronuclear formation when injected into porcine eggs (porcine-zebra, porcine-horse, and porcine-porcine ICSI embryos) and we evaluated SMARCA4 levels in pronuclei. Three biological replicates were performed this experiment.

#### Experiment 2

Domestic horse eggs were injected with zebra sperm (Zebroid experimental group) or horse sperm (Horse experimental group). *In vitro* embryo development was evaluated until the blastocyst stage and then all embryos were fixed for immunofluorescence analysis of YAP1 and SOX2. Three biological replicates were performed for this experiment.

#### Experiment 3

SCNT embryos were produced using horse oocytes derived from abattoir ovaries. Two experimental groups were performed according to the donor cell: 1) zebra SCNT and 2) domestic horse SCNT. *In vitro* embryo development was evaluated until the blastocyst stage and all embryos were fixed for immunofluorescence analysis of YAP1 and SOX2. Three biological replicates were performed for this experiment.

### Ethics and animal welfare statement

The experiments performed in this manuscript did not required the approval from the Ethics and Animal Welfare Committee of the Faculty of Agriculture, University of Buenos Aires given that no living animals were involved (CICUAL-FAUBA, Res. CD 1476/19, Reglamento para el cuidado y uso de animales para enseñanza, investigación y servicios). Fundación Temaikén is a member of WAZA (World Association of Zoos and Aquariums) and AZA (Association of Zoos and Aquariums), and thus complies with their standards on animal welfare and ethical research protocols.

### Oocyte collection and *in vitro* maturation

Horse ovaries were obtained from the abattoir (Frigorífico Land, located in Córdoba, Argentina) during breeding season and were processed locally as described by [27]. Briefly, cumulus-oocyte complexes (COCs) were recovered by follicular scraping of all visible follicles using a surgical bone curette, with the contents washed into a 50 mL tube with Dulbecco’s phosphate-buffered saline (DPBS, 14190136, Thermo Fisher Scientific) solution. After the contents had settled to the bottom of the tube, the content was transferred with a micropipette to a petri dish and diluted with Hepes-buffered Tyrode´s medium containing albumin, lactate and pyruvate (TALP-H, [28]). COCs were isolated and washed twice in TALP-H. The COCs were kept in TALP-H at room temperature (20–25°C) and transported to the laboratory located in Buenos Aires, Argentina. Transportation time varied from 20 to 24 h. *In vitro* maturation was performed for 22–24 h in 100-μL drops of bicarbonate-buffered Tissue Culture Medium (TCM-199, 11150–059, Thermo Fisher Scientific) supplemented with 10% v/v fetal bovine serum (FBS, SH30406.02IH25, GE Healthcare Life Sciences, HyCloneTM, USA), 1 μL/mL insulin-transferrin-selenium (ITS, 51300044, Thermo Fisher Scientific), 1 mM Na pyruvate (P2256), 100 mM cysteamine (M9768), 10 μg/mL Follicle Stimulating Hormone (FSH, NIH-FSH-P1, Folltropin^®^, Bioniche, Ontario, Canada), and 1% v/v penicillin-streptomycin antibiotic (ATB, P4458)under 300 μL mineral oil (M8410) in 5% CO_2_ in humidified air at 38.5 °C. Between 20 and 30 COCs were placed in each drop.

Porcine COCs were aspirated from ovaries derived from the local slaughterhouse (Frigorífico Minguillon, registration number 1466, SENASA) using an 18-gauge needle attached to a 10 mL disposable syringe. Compact COCs were selected, washed twice in TALP-H and matured for 44 h in 100-μL drops of TCM-199 under mineral oil, supplemented with 0.3 mM sodium pyruvate, 100 mM cysteamine, 5 μg/mL myo-Inositol (I5125), 1 μL/mL ITS and 1% v/v ATB, 10 μg/mL FSH, 5 ng/mL Fibroblast Growth Factor (F3685) and 10% v/v porcine follicular fluid. Between 20 and 30 COCs were placed in each drop. Follicular fluid was obtained from follicles of 3 to 6 mm of diameter, centrifuged at 400 g for 30 min at 5°C, filtered and then aliquoted and stored at -20°C.

### Cumulus cell and zona pellucida removal

The cumulus cells of both horse and porcine COCs were removed by vortexing for 3 min in TALP-H containing 3 mg/mL hyaluronidase (H3506). For experiment 1 and 2, matured zona-intact porcine and horse oocytes, respectively, were subjected to ICSI. For experiment 3, the zona pellucida of matured horse oocytes was removed by incubation in 1.5 mg/mL pronase (P8811) in TALP-H on a warm plate at 38.5°C. Zona pellucida-free oocytes (ZF-oocytes) were washed in TALP-H and maintained in a 50-μL drop of DMEM/F12 supplemented with 5% v/v FBS and 1% v/v ATB until enucleation.

### ICSI and embryo culture

Frozen sperm cells from a plain male zebra, frozen semen from a fertile stallion, and porcine refrigerated semen were used for ICSI experiments. After the dead by natural causes of a male zebra at the Fundación Temaikén zoo, sperm cells from the epididymis were collected and frozen by the authors for gene banking. Up to date, these samples were only used for this study. Horse frozen semen was donated by the artificial insemination horse center located in Buenos Aires, Argentina. Porcine semen was donated by “Agroceres PIC” located in Buenos Aires, Argentina. For frozen sperm, a portion of one straw was cut under liquid nitrogen and submerged in 4 mL of TALP-H, centrifuged twice at 300 x *g*, washing with the same solution. A 1-μL aliquot of semen was taken from the supernatant and placed in a 3-μL drop of 7% v/v polyvinylpyrrolidone (Irvine Scientific, Santa Ana, CA, US) in TALP-H. ICSI was performed using a 7 μm (for horse/zebra sperm) or 9 μm (for porcine sperm) glass sharp micropipette in an inverted microscope (Nikon Eclipse TE-300 microscope Nikon, Melville, NY, USA) using hydraulic micromanipulators (Narishige, Medical Systems, Great Neck, NY, USA). Presumptive zygotes were cultured in 50% DMEM F12/ 50% Global Total^®^ (LGGT-030, LifeGlobal, Guilford, CT, USA) with 6% v/v FBS and 1% v/v ATB for up to eleven days. Cleavage was assessed on day 5 and the blastocyst rate was recorded form day 7, daily until day 11, given horse ICSI blastocysts are reported to appear *in vitro* up to day 10 [29]. All day 7–8 ICSI blastocysts were fixed for immunofluorescence. For evaluation of pronuclear (PN) formation in porcine oocytes, presumptive zygotes were in cultured in porcine zygote medium for 16–18 h and then centrifuged for 10 min at 1000 x *g* and fixed. After immunofluorescence, embryos were classified according to the presence of pronuclei: two PN (2-PN), one PN with the presence of a semi-condensed or condensed sperm (1-PN), or a semi-condensed or condensed sperm with no evidences of PN (No activation). Embryos that exhibited a different pattern were included into “other” category.

### Enucleation, nuclear transfer and embryo construction

ZF-oocytes were incubated for 5 min in DMEM/F12 with 5% v/v FBS, 1% v/v ATB and 0.01 mg/mL Hoechst 33342 dye (B2261). The metaphase plate was removed by aspiration using a blunt pipette under ultraviolet light and a closed holding pipette to manipulate the oocyte. Enucleation was performed using hydraulic micromanipulators (Narishige, Medical Systems, Great Neck, NY, USA) mounted on a Nikon Eclipse TE-300 microscope in a 100-μL drop of TALP-H supplemented with 0.5 μg/mL cytochalasin B (C6762). ZF-enucleated oocytes were kept in DMEM/F12 with 5% v/v FBS and 1% ATB v/v until NT. ZF-enucleated oocytes were transferred individually to a 50-μL drop of DMEM/F12 containing 1 mg/mL phytohemagglutinin (L8754). After a few seconds, oocytes were dropped over a single donor cell resting in a 100-μL drop of TALP-H. The couplets were then placed in fusion medium [0.3 M mannitol (M9647), 0.1 mM MgSO_4_ (63138), 0.05 mM CaCl_2_ (C7902) and 1 mg/mL polyvinyl alcohol (P8136)] for 2–3 min before being placed in a fusion chamber containing 2 mL fusion medium at 38.5°C. Fusion was performed using BTX Electro-Cell Manipulator 830 (BTX, Inc., San Diego, CA). The fusion parameters were as follows: double-direct current pulse of 1.2 kV/cm, for 30 μs and 0.1 s apart. Couplets were then individually placed in a 5-μL drop of DMEM/F12 with 5% FBS v/v and 1% v/v ATB and incubated under mineral oil at 38.5°C in 5% CO_2_ in air. Each couplet was assessed 10 to 20 min after the pulse. Absence of the donor cell in the drop confirmed fusion. Non-fused couplets were fused again. Two hours after fusion, ZF-reconstructed embryos (ZFREs) were activated. ZFREs were treated with 8.7 mM ionomycin (I24222, Thermo Fisher Scientific) in TALP-H for 4 min, followed by individual culture for 4 h in a 10-μL drop of DMEM/F12 supplemented with 5% v/v FBS, 1% v/v ATB, 1 mM 6-dimethylaminopurine (D2629) and 5 μg/mL cycloheximide (C7698, [30]).

### *In vitro* embryo culture of SCNT embryos

A modified well-of-the-well system was used to *in vitro* culture ZFREs [31]. The microwells were produced by gently pressing a heated glass capillary into the base of a 35 x 10 mm Petri dish. Microwells were covered with a 50-μL drop of DMEM/F12 containing 5% v/v FBS, 1% v/v of ATB and 1 μL/mL ITS. Taking advantage of embryo aggregation [32, 33] two ZFREs were randomly introduced into each microwell for experiment 3 in all experimental groups. The number of ZFREs per drop was similar between groups. Embryos were cultured in a humidified gas mixture of 5% CO_2_, 5% O_2_ and 90% N_2_ at 38.5°C. Cleavage was assessed on day 5 and the blastocyst rate was recorded form day 7, daily until day 11. All day 7–8 cloned blastocysts were fixed for immunofluorescence.

### Cell culture

Adult fibroblasts were obtained after culture of minced tissue from neck skin biopsies from an adult male plain zebra after death at Fundación Temaikén, Escobar, Buenos Aires, Argentina. Adult fibroblasts from a death male horse were used for horse SCNT. Cells were cultured in Dulbecco’s modified Eagle’s medium (DMEM/F12, 11320033, Thermo Fisher Scientific, Waltham, MA, USA) with 10% v/v FBS, 1% v/v ATB, and 1 μL/mL ITS in 5% CO_2_ in humidified air at 38.5°C. Once the primary culture was established, fibroblasts were subcultured every 4 to 6 days, cryopreserved in DMEM with 10% v/v FBS and 10% v/v dimethyl sulfoxide (DMSO, D2650), and stored in liquid nitrogen until used. Quiescence of donor cells was induced by growth to confluency for 3 to 5 days prior to nuclear transfer. Populations of cells were prepared by trypsinization (25200056, Thermo Fisher Scientific). Cells were then washed and resuspended in DMEM/F12.

### Embryo fixation, immunofluorescence and cell counting

Presumptive ICSI zygotes, and SCNT and ICSI blastocysts were fixed for 20 min in 4% formaldehyde (47608) in DPBS, rinsed in DPBS with 0.4% w/v bovine serum albumin (BSA, A6003), and stored at 4°C in 96-well plates. Embryos were treated with permeabilization solution [DPBS containing 0.2% v/v Triton X-100 (21123)] for 15 min and washed in blocking buffer [DPBS containing 0.1% v/v Tween 20 (P9416) and 0.4% w/v BSA]. Presumptive zygotes fixed 18–20 h after ICSI on porcine oocytes were incubated with SMARCA4 antibody [Brg-1 (G-7), 1:100, SC-17796, mouse monoclonal, Santa Cruz Biotechnology, Dallas, TX, USA. AB_626762] overnight at 4°C in blocking buffer. A negative control group without primary antibody was included for all assays. After washing, all embryos were incubated with secondary antibody (Alexa Fluor^®^ Plus 594,1:1000, donkey anti-mouse, #A32744, Thermo Fisher Scientific) in blocking buffer for 1 h in the dark. Fixed blastocysts were incubated with SOX2 antibody [Sox-2 (Y-17), 1:200, SC-17320, goat polyclonal, Santa Cruz Biotechnology, AB_2286684] overnight at 4°C in blocking buffer. Afterwards, embryos were washed three times for 15 min in blocking buffer, followed by 1 h incubation at room temperature with YAP1 antibody [YAP (H-9), 1:100, SC-271134, mouse monoclonal, Santa Cruz Biotechnology, AB_10612397]. After washing, embryos were incubated with secondary antibodies (Alexa Fluor^®^ 555, 1:1000, donkey anti-goat, # 21432 and Alexa Fluor^®^ 488, 1:1000, donkey anti-mouse IgG #A21202, Thermo Fisher Scientific) in blocking buffer for 1 h in the dark. Finally, presumptive zygotes and blastocysts were mounted in Vectashield^®^ containing 1.5 μg/mL DAPI (Vector Laboratories, Burlingame, CA), and slides were scanned using an inverted confocal microscope (Olympus IX83 Spinning Disk Confocal System). The analysis of total cell number, and quantification of SOX2 positive (SOX2+) and YAP1 positive (YAP1+) nuclei was performed manually with FIJI image processing software. Intensity of SMARCA4 protein in PN was only analyzed in ICSI zygotes with the presence of 2 PN. A region of interest was drawn around each pronucleus and the average pixel intensity was determined with FIJI image processing software [34].

### Statistical analyses

All statistical analyses were performed using GraphPad Prism software. Comparisons of two groups were performed using Mann-Whitney U test. Comparisons of three groups were performed using Kruskal-Wallis test, with Dunn’s multiple comparisons test. Embryo preimplantation development rates were compared using two-tailed Fisher’s exact test with a CI of 95%. Differences were considered statistically significant with a value of P ≤ 0.05.

## Results

### Experiment 1: Pronuclear formation and SMARCA4 recruitment after injection of zebra, horse and porcine sperm into porcine oocytes

**Fig 1:**
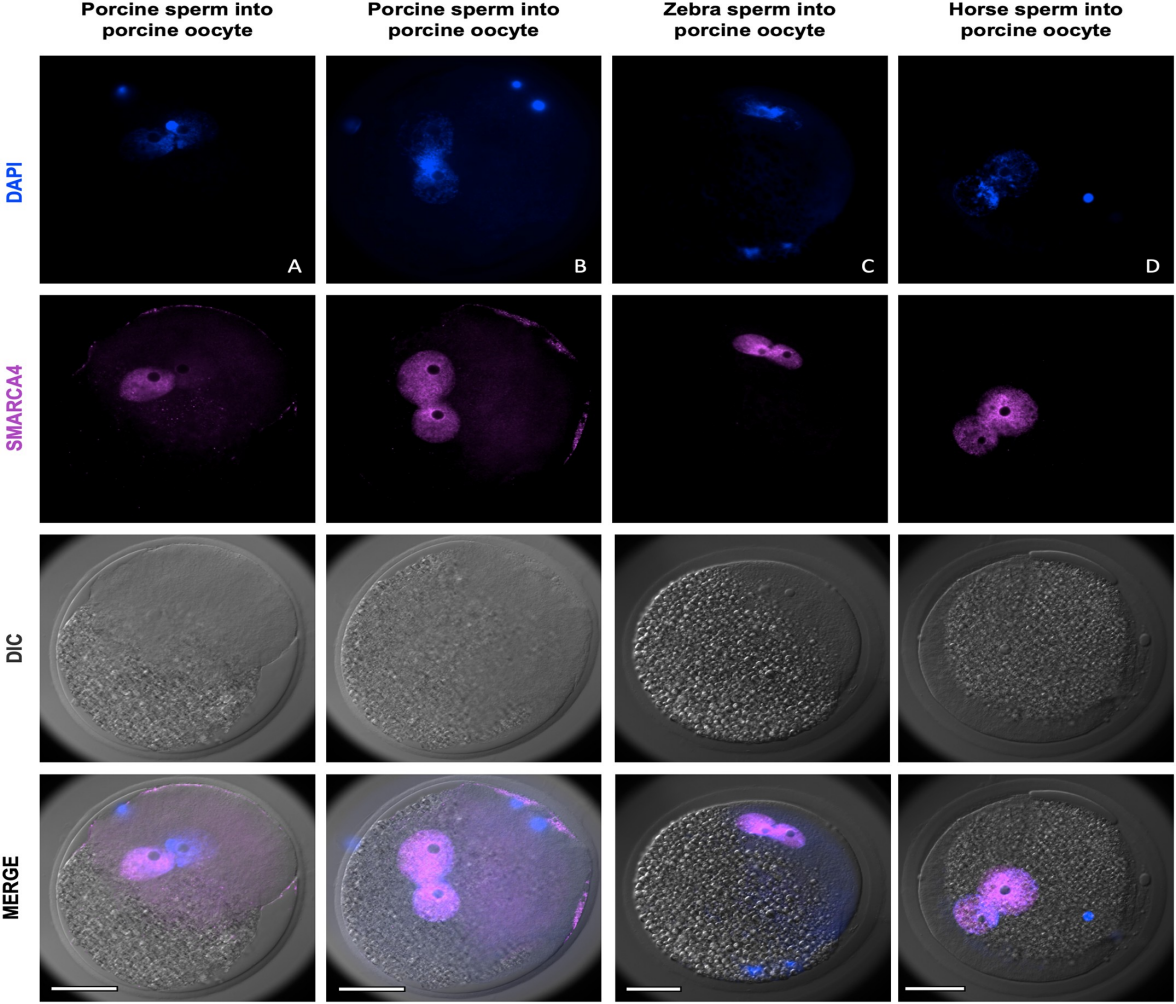
Representative immunofluorescent staining of ICSI zygotes of the indicated groups. **(A)** Homospecific porcine-porcine zygote showing asymmetric SMARCA4 levels between PN. **(B)** Homospecific porcine-porcine zygote showing similar SMARCA4 levels between PN. **(C)** Heterospecific zebra-porcine zygote. **(D)** Heterospecific horse-porcine zygote. Scale bars indicate 50 μm.

**Fig 2:**
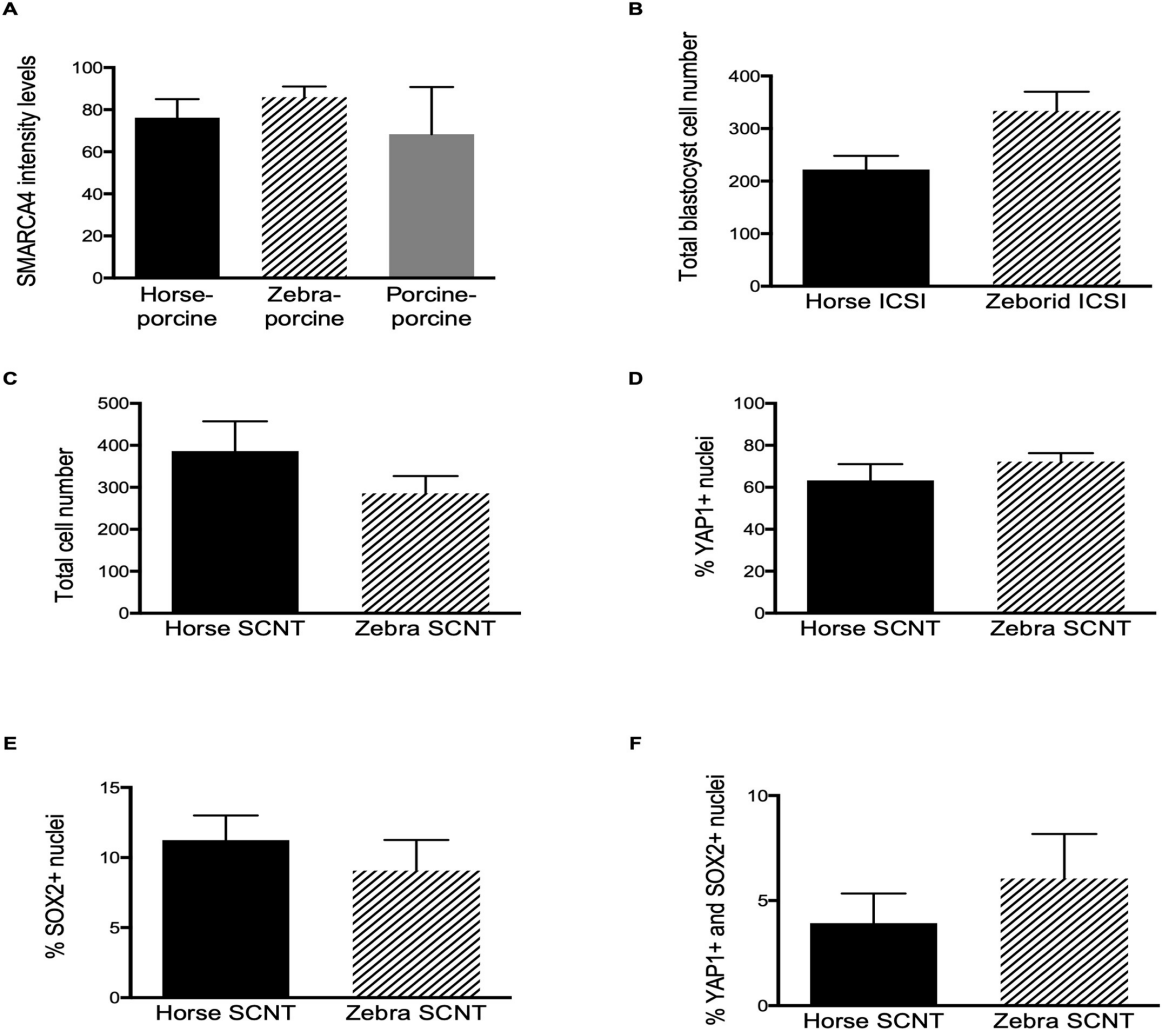
Analysis of embryo quality and SMARCA4, YAP1 and SOX2 expression. **(A)** Total cell number in the blastocysts from the indicated groups. **(B)** Percentage of YAP1-positive (YAP1+) nuclei in blastocysts from the indicated groups. **(C)** Percentage of SOX2-positive (SOX2+) nuclei in blastocysts from the indicated groups. **(D)** Percentage of YAP1+ and SOX2+ nuclei in blastocysts from the indicated groups. **(E)** Immunofluorescence quantification (arbitrary units) of SMARCA4 in ICSI zygotes of the indicated groups. **(F)** Total cell number in ICSI blastocysts from the indicated groups. Errors bars in all panels display standard error of the mean (SEM).

To first assess whether zebra semen was able to induce egg activation, we injected zebra spermatozoa into matured porcine oocytes for detection of PN formation and analysis of SMARCA4 protein expression. As control groups, horse and porcine sperm cells were also injected. Zebra sperm were capable of inducing porcine egg activation, and no differences were observed in the number of oocytes with 2-PN among experimental groups (Table 1) although the number of non-activated eggs was lower in zebra group compared with the domestic horse (p = 0.0013) and the porcine (p = 0.0365). SMARCA4 was found to be localized in both PN in all interspecific experimental groups, and it was absent in polar bodies, metaphase plate or condensed sperm (Fig 1). Interestingly, out of 16 homospecific porcine 2-PN-ICSI zygotes analyzed, 31.25% showed clear asymmetric intensity levels of SMARCA4 between PN, with only one SMARCA4 positive (Fig 1A). The rest of the porcine-porcine zygotes (68.75%) showed similar SMARCA4 intensity levels between PN (Fig 1B). All interspecific zebra/horse-porcine zygotes showed similar intensity levels in both PN. We next compared SMARCA4 pronuclear intensity levels among groups, but considering only those embryos with similar SMARCA4 levels between PN. Non-significant differences were found in SMARCA4 intensity levels in this comparison (Fig 2A). These results demonstrated that zebra sperm induce porcine egg activation and that maternal SMARCA4 can be recruited to a heterologous pronucleus at similar levels than the counterpart pronucleus.

**Table 1:** Pronuclear evaluation of zebra, horse and porcine sperm cells injected into matured porcine oocytes.

Group	# presumptive zygotes	# 2-PN (%)	# 1-PN (%)	# No activation (%)	# Other (%)
Zebra-porcine	30	9 (30.00)	1 (3.33)^a^	17 (56.66)^a^	3 (10)
Horse-porcine	31	14 (45.16)	9 (29.03)^b^	5 (16.12)^b^	2 (6.45)
Porcine-porcine	47	16 (34.04)	13 (27.65)^b^	15 (29.78)^b^	6 (12.79)
Total	108	39 (36.11)	23 (21.29)	37 (34.25)	11 (10.18)

Different superscript letters indicate statistical significance. (Fisher’s exact test, P-values < 0.05). PN: pronucleus.

### Experiment 2: Intragenus ICSI zebra hybrid embryos

#### In vitro *development of zebroid ICSI embryos*

After zebra semen evaluation in experiment 1, we evaluated whether zebra sperm had the ability to induce domestic horse egg activation and trigger *in vitro* embryo development to blastocyst stage, we carried out ICSI using domestic horse sperm (control group) or zebra sperm. Zebra sperm showed a slightly lower ability to trigger embryo development compared to horse sperm revealed by the lower cleavage rate (p = 0.01). Remarkably, cleaved zebroid embryos developed to blastocyst stage at similar rates compared to the control group (Table 2). Altogether, these results showed that sperm cells recovered from the epididymis of a zebra can successfully induce domestic horse egg activation and trigger embryo development to blastocyst stage.

**Table 2:** *In vitro* preimplantation development of zebroid and horse ICSI embryos.

Group	# ICSI embryos	# cleaved embryos (%)	# day 8 blastocysts (%)	# day 11 blastocysts (%)
Horse	75	50 (66.66)^a^	9 (12.00)	13 (17.33)
Zebroid	49	21 (42.85)^b^	2 (4.08)	3 (6.12)
Total	124	71 (57.25)	11 (8.87)	16 (12.94)

Different superscript letters indicate statistical significance. (Fisher’s exact test, P-value < 0.05).

#### Zebroid ICSI embryo cell early differentiation

**Fig 3:**
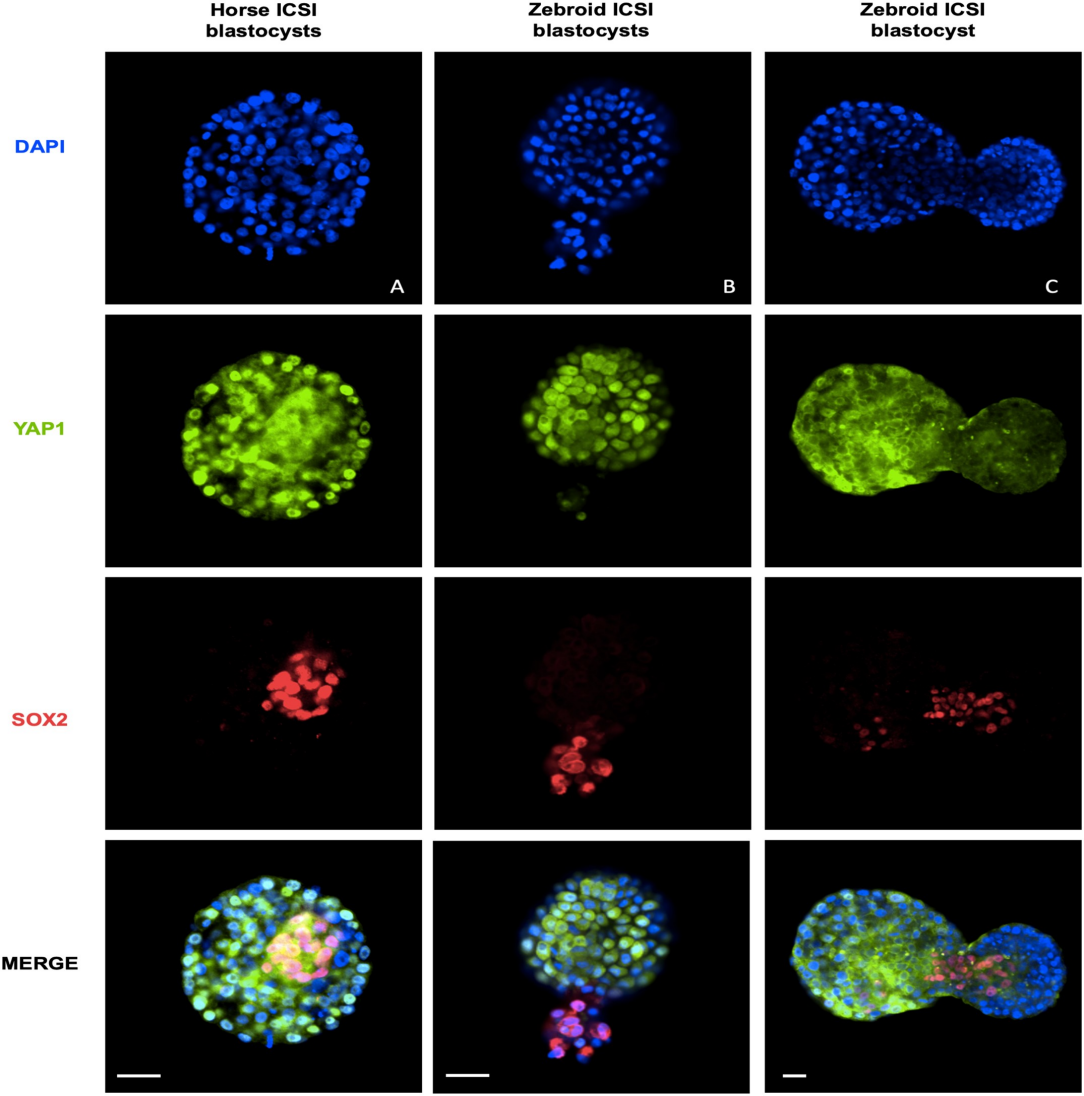
Representative immunofluorescent staining of ICSI blastocyst stage embryos of the indicated groups. Scale bars indicate 50 μm.

To study horse and zebroid blastocysts early cell differentiation of the ICM and the trophectoderm (TE), we evaluated total cell number and YAP1 and SOX2 expression patterns in zebroid and horse ICSI blastocysts. A total of 6 embryos were analyzed in this study, 3 for each experimental group. The majority of the nuclei were positive for YAP1, and most SOX2+ nuclei were grouped in a particular area of the blastocyst (putative ICM cells) and were found negative for YAP1 (Fig 3A and 3B). No differences were observed in total blastocyst cell number (Fig 2B). Interestingly, one day 11 hatched zebroid blastocyst had a different YAP1 expression pattern (Fig 3C). This embryo, with higher cell number and an advanced developmental stage, showed a lower percentage of YAP1+ nuclei. These results suggest that heterospecific reprogramming of zebra sperm cells by domestic horse oocytes might not affect the localization of YAP1 and SOX2 compared to the homospecific counterpart.

### Experiment 3: Intragenus SCNT zebra embryos

#### In vitro development of SCNT zebra embryos

To study whether domestic horse enucleated oocytes support zebra SCNT embryo development, we compared *in vitro* development up to the blastocyst stage of homospecific horse cloned embryos with heterospecifc intragenus zebra embryos. Although no differences in cleavage rates between groups were observed, cloned zebra blastocyst rate was significantly higher on day 8 (p = 0.0151) and on day 11 (p = 0.0237) compared to the control (Table 3). These results demonstrated that *in vitro* matured horse oocytes support the development of zebra SCNT embryos up to the blastocyst stage without compromising developmental rates.

**Table 3:** *In vitro* preimplantation development of zebra and horse SCNT embryos.

Group	# ZFREs	# cleaved ZFREs (%)	# day 8 blastocysts (%)	# day 11 blastocysts (%)
Horse SCNT	55	41 (74.54)	5 (9.09)^a^	7 (12.72)^a^
Zebra SCNT	58	44 (75.86)	16 (27.58)^b^	18 (31.03)^b^
Total	113	85 (75.22)	21 (18.54)	25 (22.12)

Different superscript letters indicate statistical significance. (Fisher’s exact test, P-values < 0.05). ZFREs, zona pellucida-free reconstructed embryos.

#### SCNT zebra embryo early cell differentiation

**Fig 4:**
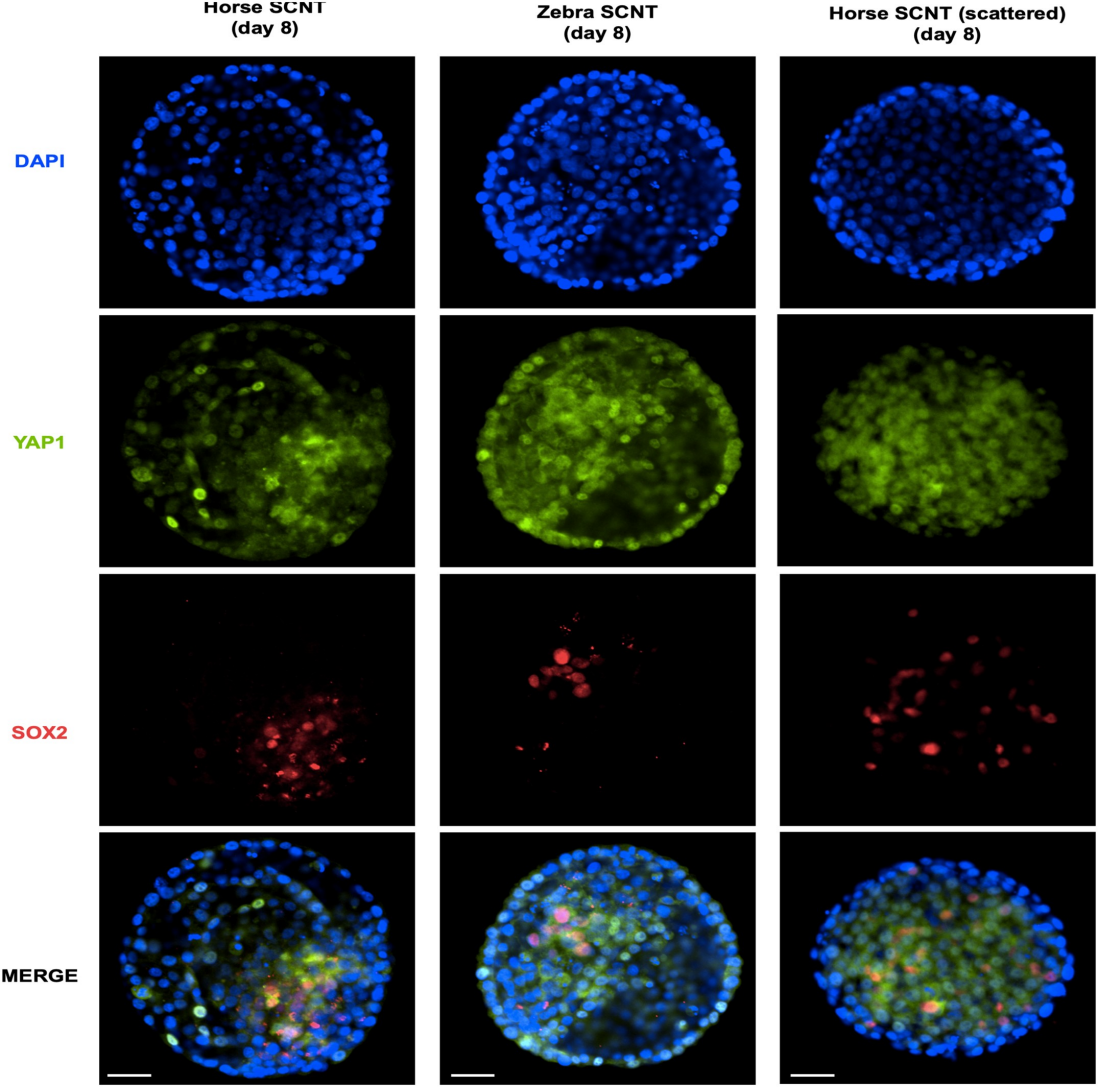
Representative immunofluorescent staining of SCNT blastocyst stage embryos of the indicated groups. Scale bars indicate 50 μm.

To investigate whether zebra SCNT blastocysts could have a compromised early cell differentiation of ICM and TE due to potential failures during heterospecific epigenetic reprogramming, we performed immunofluorescence analysis to study YAP1 and SOX2 protein expression patterns. A total of 14 SCNT blastocysts were analysed, 9 from zebra and 5 from horse experimental groups. Overall, non-significant differences in total cell number, or in number of SOX2+ or YAP1+ nuclei were observed between groups (Figs 2C–2E and 4). As observed for ICSI embryos, in SCNT blastocyst the majority of the nuclei were positive for YAP1, and most SOX2+ nuclei were grouped in a particular area of the blastocyst and were found negative for YAP1. Interestingly, one blastocyst of each group showed a scattered expression pattern of SOX2 without a clear grouped arrangement and with an increased number of cells positive to both SOX2 and YAP1 (Figs 2F and 4). These observations suggest that heterospecific reprogramming of zebra skin cells by domestic horse oocytes does not significantly affect lineage specification compared the its homospecific counterpart.

## Discussion

Taking advantage of remarkably similar morphology and ecological niches among equids [35] domestic horses can be postulated as a model to optimize ART for its future application in wild equid conservation programs [36]. To the best of our knowledge, the data reported here demonstrate for the first time that the domestic horse oocyte supports *in vitro* preimplantation embryo development of another species of the genus *Equus*, the zebra, without compromising the expression and localization of the early cell differentiation markers, YAP1 and SOX2, or blastocyst cell number.

In endangered species, there is a limited supply of cryopreserved semen creating a barrier in the application of reproductive biotechnologies in conservation breeding programs. Therefore, developing *in vitro* methods for estimating *in vivo* fertilization rates of cryopreserved sperm are needed. Embryo production through ICSI overcomes limited semen availability, and our results demonstrate that the ability of zebra sperm cells to produce embryos can be tested by producing *in vitro* hybrid (zebroid) embryos. Testicular and epididymal sperm for ICSI has been successfully used in humans as an alternative from ejaculated sperm in patients with cryptozoospermia [37, 38] and no increased risk in neonatal outcomes was found in newborns [39]. Moreover, in mice, caput sperm have the same potential to produce offspring after ICSI than cauda sperm [40]. These previous findings demonstrated that the changes that occur during the transit through the epididymis might not be crucial for normal fertilization and embryo development after ICSI. Using a porcine model, we showed that epididymal zebra sperm cells induce oocyte activation and recruit SMARCA4 during pronuclear formation. Moreover, the translocation of SMARCA4 into the pronuclei could be independent of the genetic background of the injected sperms given that horse sperm cells were also able to recruit same levels of SMARCA4. Our study represents the first looking at SMARCA4 recruitment during heterospecific reprogramming of male nuclei and it could be used as an interesting approach to identify chromatin remodeling failures during heterospecific reprogramming. Remarkably, the higher proportion of non-activated oocytes observed with zebra sperm in porcine eggs was reflected by the lower cleavage rate observed in zebroid ICSI embryos in experiment 2. Thirty percent of the homospecific porcine ICSI zygotes showed a remarkable difference of SMARCA4 recruitment between PN, and since SMARCA4 recruitment during pronuclear development is likely crucial for later embryo survival [22], this observation might be linked to the lower developmental rates reported for porcine ICSI embryos when no additional artificial stimulation is applied [41].

Heterospecifc SCNT using phylogenetically related (intragenus) or distant species has been attempted in numerous mammalian species, including endangered animals, with variable results [42–45]. Furthermore, heterospecific cloning has been attempted to rescue extinct subspecies such as Pyrenean ibex [46]. In horses, we have previously reported that felid oocytes in interspecies SCNT were more prone to support preimplantation embryo development of a horse somatic cell than bovine or porcine oocytes [47], possibly due to a closer phylogenetic proximity of felid species to horses [48]. Interestingly, the first equid produced by SCNT was an equid hybrid, a mule [49]. Supporting this finding, a recent study demonstrated that horse ooplasm supports somatic cell reprograming of a mule without compromising H19 gene imprinting [50]. In contrast to zebras, mules share half of the genetic background and the mitochondrial DNA with domestic horses, which could facilitate nuclear reprogramming and embryo developmental competence. Our findings demonstrate that the domestic horse oocyte supports *in vitro* development of zebra SCNT embryos up to the blastocyst stage. Surprisingly, the blastocyst developmental rate obtained from the zebra SCNT group was significantly higher compared to the homospecific SCNT. However, this could be due to a fibroblast primary culture effect as it influences the developmental success of SCNT [30, 32]. In the early 1980s, the remarkable plasticity of equine pregnancy was revealed when embryos of Przewalski’s horse, domestic horse, domestic donkey, and Grant’s zebra were transferred to horse, donkey and mule recipients to obtain live offspring [1, 3, 4]. Thus, domestic equid females could be used as recipients for SCNT or ICSI zebra embryos, or potentially any other *in vitro* produced wild equid embryo, opening the possibility to deeply investigate the amazing features of equine pregnancy such as immunological tolerance, placentation, and pregnancy-related endocrinological functions.

The low efficiency of SCNT has been primarily attributed to failures during nuclear reprogramming processes, which are necessary to restore the totipotency of the donor somatic cell nucleus [51, 52]. In horses, several strategies were recently attempted to improve nuclear reprogramming, but non-significant differences were observed in development [53]. Moreover, failures during cell reprogramming could affect the expression pattern of proteins involved in cell-fate specification and compromise embryo viability [54]. The Hippo/YAP signaling cascade plays an important role in early embryo cell differentiation [55]. In the TE, YAP translocates into the nucleus to activate TE-specific genes, whereas in the ICM, YAP is phosphorylated leading to its cytoplasmic sequestration and extrusion from the nucleus [56]. Furthermore, SOX2 was reported to be one of the earliest known unique markers of ICM progenitors [25]. In our study, we have not found significant differences between domestic horse and zebra embryos YAP1 and SOX2 expression patterns, suggesting that domestic horse oocytes are capable of supporting *in vitro* preimplantation embryo development of an embryo with either half or the entire zebra genome of a without compromising the HIPPO cascade. We have found that SOX2 and YAP1 are mutually exclusive in nuclei of equid blastocysts as it was previously reported in mice [25]. Interestingly, one cloned blastocyst from each experimental group showed an altered expression pattern of YAP1/SOX2. Since differentiation of ICM and TE is crucial for later embryo survival, we hypothesized that these embryos could had a compromised developmental competence and that evaluation of these proteins could be useful for assessing embryo quality in horses.

Up to the present, the only reproductive strategy used to preserve wild equids is by conservation breeding programs. Our results show that advanced ARTs, such as ICSI and intragenus SCNT, are feasible to be applied for the preservation of endangered or resurrection of wild zebras such as the *Equus quagga quagga*. Also, our data strongly suggest that the HIPPO signaling pathway, during early embryo cell differentiation, is conserved in equids as it was reported for mice [25] and cattle [57] and support the use of these markers to identify cell fate on equine blastocyst [58].
